# Preferential Biological Processes in the Human Limbus by Differential Gene Profiling

**DOI:** 10.1371/journal.pone.0061833

**Published:** 2013-04-22

**Authors:** Martin N. Nakatsu, Lily Vartanyan, Daniel M. Vu, Madelena Y. Ng, Xinmin Li, Sophie X. Deng

**Affiliations:** 1 Cornea Division, Jules Stein Eye Institute, University of California Los Angeles, Los Angeles, California, United States of America; 2 Department of Pathology and Laboratory Medicine, David Geffen School of Medicine, University of California Los Angeles, Los Angeles, California, United States of America; Johns Hopkins University, United States of America

## Abstract

Corneal epithelial stem cells or limbal stem cells (LSCs) are responsible for the maintenance of the corneal epithelium in humans. The exact location of LSCs is still under debate, but the increasing need for identifying the biological processes in the limbus, where LSCs are located, is of great importance in the regulation of LSCs. In our current study we identified 146 preferentially expressed genes in the human limbus in direct comparison to that in the cornea and conjunctiva. The expression of newly identified limbal transcripts endomucin, fibromodulin, paired-like homeodomain 2 (PITX2) and axin-2 were validated using qRT-PCR. Further protein analysis on the newly identified limbal transcripts showed protein localization of PITX2 in the basal and suprabasal layer of the limbal epithelium and very low expression in the cornea and conjunctiva. Two other limbal transcripts, frizzled-7 and tenascin-C, were expressed in the basal epithelial layer of the limbus. Gene ontology and network analysis of the overexpressed limbal genes revealed cell-cell adhesion, Wnt and TGF-β/BMP signaling components among other developmental processes in the limbus. These results could aid in a better understanding of the regulatory elements in the LSC microenvironment.

## Introduction

The preservation of corneal transparency and maintenance of the corneal epithelium are critical for proper visual function. The corneal surface itself is protected by non-keratinized stratified squamous epithelial cells. Due to constant cell turnover, these corneal epithelial cells are thought to be replenished by a reservoir of corneal epithelial stem cells or limbal stem cells (LSCs) at the limbus [1], [2]. It has been proposed that LSCs reside at the basal layer of the limbus [3], [4], which is a highly vascularized structure at a transitional zone that separates the cornea and conjunctiva. Ocular injuries, as a result of chemical burns or various ocular diseases, can lead to limbal stem cell deficiency (LSCD), which is a pathological condition due to LSC depletion and/or destruction of the stem cell niche.

Like all other adult stem cells, LSCs are thought to be slow cycling and undergo infrequent division in vivo [4], but have the potential for self-renewal, rapid proliferation and differentiation under the proper conditions, such as in wound healing or in tissue culture [5]–[7]. LSCs give rise to transient amplifying cells (TACs) that continue to proliferate at a limited capacity and migrate centripetally and apically toward the central cornea to help maintain the normal homeostasis of the corneal epithelium [8]. Due to the heterogeneous cell population of the limbus, which include TACs, terminally differentiated cells and a low number of stem cells in the limbus [9], identification of a specific LSC marker has proven to be very challenging. Two of the more well known markers are ATP-binding cassette sub-family G member 2 (ABCG2) and ΔNp63α. Both have been shown to be highly expressed at the basal and suprabasal layers of the limbus in numerous studies with little to no expression in the central and peripheral cornea [10]–[13]. Other identified LSC putative markers include N-cadherin, CCAAT-enhancer-binding protein Δ (C/EBPΔ), keratin (K) 15, integrin α9 and α-enolase [10], [14]–[18].

Despite highly localized expression in the limbal basal and suprabasal epithelium and limited expression in the cornea, many of these molecules, such as ABCG2, ΔNp63α and N-cadherin are expressed in the conjunctiva as well [9], [13], [19]. Recently, numerous mammalian gene profiling studies have examined differential gene expression in various ocular epithelia [16], [19]–[25]. However, no such study has compared all three ocular surface structures in humans as of yet.

To understand the unique microenvironment of LSCs, we performed a microarray analysis of the epithelium along with the immediate underlying stroma from the conjunctiva, cornea and limbus. We specifically included the immediate underlying stroma to identify potential LSC niche factors. We identified 146 genes that were preferentially expressed in the limbus. Gene ontology and network analyses identified several important stem cell related processes including several genes involved in two key signaling pathways, the Wnt and TGF-β/BMP pathways.

## Materials and Methods

### Human Sclerocorneal Tissue

Human sclerocorneal tissues of healthy donors were obtained from the Lions Eye Institute for Transplant and Research (Tampa, FL), the Tissue Bank International (Baltimore, MD) or the San Diego Eye Bank (San Diego, CA). The experimental protocol was evaluated and exempted by the UCLA Institutional Review Board. Only those tissues that had intact conjunctival, corneal and limbal epithelia were selected for this study. The ages of the donors ranged from 20 to 65 years. For the purpose of RNA isolation, the death to preservation time was less than 7 hours. For immunohistochemistry, death to preservation time was less than 10 hours.

### RNA Extraction and qRT-PCR

The conjunctival, corneal and limbal epithelia along with their adjacent 1/3 stroma were dissected from sclerocorneal tissues as described previously [19]. To ensure complete homogenization of tissue samples, a serrated homogenizer (Omni International, Marietta, GA) was used. Total RNA from tissues was extracted with a Qiagen RNeasy Mini Kit (Qiagen, Valencia, CA). The quantity and quality of total RNA were assessed by a NanoDrop 1000 spectrophotometer (NanoDrop, Wilmington, DE) and a 2100 Bioanalyzer (Agilent Technologies, Santa Clara, CA). Only those samples that had a RNA integrity number (RIN) >9 and exhibited minimal RNA degradation were used for subsequent experiments. The quality of the RNA was confirmed through electropherogram and nanogel analysis from the Bioanalyzer (Fig. S1).

Total RNA was reverse-transcribed using Superscript II RNase H2 reverse transcriptase (RT) (Life Technologies, Grand Island, NY) according to the manufacturer’s recommendations. The relative abundance of transcripts was detected through qRT-PCR by using a Brilliant SYBR Green qRT-PCR Master Mix (Agilent Technologies, Santa Clara, CA). The protocol used a Agilent Mx3000p real-time PCR system. Cycling conditions were as follows: an initial denaturing step of 5 minutes (m) at 94°C and subsequent 40 cycles of amplification in which each cycle consisted of 15 seconds (s) at 94°C, 30 s at 55°C and 30 s at 72°C. To generate a dissociation curve after the amplification cycles, each sample was incubated at 95°C for 1 m followed by a melting curve program (55–99°C with a 5 s hold at each temperature). The fluorescence intensity of each sample was normalized in relation to that of the housekeeping gene, glyceraldehyde-3-phosphate dehydrogenase (GAPDH). At least three independent experiments were performed on each donor and a total of 3 donors were used. The primers used for qRT-PCR are listed in Table S1.

### Microarray Hybridization and Data Analysis

Microarray methods were described previously [19], [26]. Briefly, Affymetrix U133 plus 2.0 human expression arrays (Affymetrix, Santa Clara, CA) were used and performed by the UCLA DNA Microarray Core following the standard Affymetrix GeneChip Expression Analysis protocol**.** The acquisition of array images was undertaken by using Affymetrix GeneChip Command Console 1.1 (AGCC). Subsequent raw data were analyzed using dChip software [27] with the.CEL files obtained from AGCC. We used a PM/MM difference model for estimating gene expression levels and RMA quantile approach for data normalization. Thresholds for selecting significant genes were set at a relative difference ≥2-fold, absolute difference >100 signal intensity units and p<0.05. Genes that met all three criteria were considered as significant. The acceptable false discovery rate was 5%. Analysis was performed using the Partek Genomics Software Suite (Partek, St. Louis, MI)**.** Gene ontology analysis, global functional analyses, network analyses and canonical pathway analyses were performed using the database for annotation, visualization and integrated discovery (DAVID) database, the Kyoto Encyclopedia of Genes and Genomes (KEGG) Pathway Database (Kanehisa Laboratories, Kyoto, Japan) and the Ingenuity Pathway Analysis website (Ingenuity Systems, Redwood City, CA). We have deposited the raw data at Gene Expression Omnibus (GEO) under accession number GSE38190 and we can confirm all details are Minimum Information About a Microarray Experiment (MIAME) compliant.

### Immunohistochemistry

The human sclerocorneal tissues obtained from eye banks were cut into four quadrants and embedded in OCT (Sakura Finetek, Torrance, CA) on dry ice. Tissues were cut into 8 µm sections using a Leica CM3050S cryostat (Leica Microsystems, Wetzlar, Germany) and stored at −80°C. The tissue cryosections were fixed in 4% paraformaldehyde (Fisher Scientific, Hampton, NH) for 15 m and permeabilized by 0.3% Triton X-100 (Sigma-Aldrich, St. Louis, MO) in phosphate-buffered saline (PBS) (Life Technologies). Fixed slides were incubated with 5% blocking serum (Jackson ImmunoResearch Laboratories, West Grove, PA) in 1% bovine serum albumin (BSA) in PBS for 30 m, washed three times with 1% BSA/PBS and incubated with a primary antibody for 1 hour. The slides were then washed three times with 1% BSA/PBS and labeled with an appropriate secondary antibody (Life Technologies) for 1 hour. Primary and secondary antibodies used are summarized in Table S2. The nuclei were labeled with Hoechst 33342 (Life Technologies) at 0.5 µg/ml for 10 m. The slides were then washed with PBS three times and mounted in gel mount medium (Sigma-Aldrich). Pictures were taken under a 25x objective using a Zeiss fluorescent microscope (Carl Zeiss Inc., Oberkochen, Germany) and Leica TCS-SP confocal microscope (Leica Microsystems).

### Statistical Analysis

To eliminate the variation between experiments in the qRT-PCR and microarray, values were obtained from each of the three independent experiments and averaged. To allow for direct comparison between the data from the microarray experiments and that from the qRT-PCR, the gene expression values in the limbus were set to 1. The values in the other two tissue types were calculated as a ratio against that in the limbus. Bar graphs represent mean ± standard error of the mean (s.e.m.) from three separate experiments.

## Results

### Isolated RNA is Tissue Specific

Corneal, conjunctival and limbal RNA were isolated from both eyes of three healthy human donors. There were 4 replicates for the conjunctiva and limbus samples and 3 replicates for the cornea sample. The total RNA yield from one cornea was negligible and therefore not used. To further verify the tissue specificity of the samples, the expression profiles of three well known signature genes were quantified through qRT-PCR. K12, a marker for the mature cornea epithelium, was highly expressed in the cornea compared to the limbus and conjunctiva (Fig. S2). Mucin 5AC, a conjunctiva marker, was preferentially expressed in the conjunctiva with minimal expression in the cornea and limbus (Fig. S2). K15, which is expressed in the basal epithelium of both the limbus and conjunctiva, had elevated expression in the limbus and conjunctiva tissue (Fig. S2). Based on these results, the RNA isolated was specific to each tissue type as expected and of high quality for the subsequent microarray experiment.

### Gene Expression Correlation between Samples

**Figure 1:**
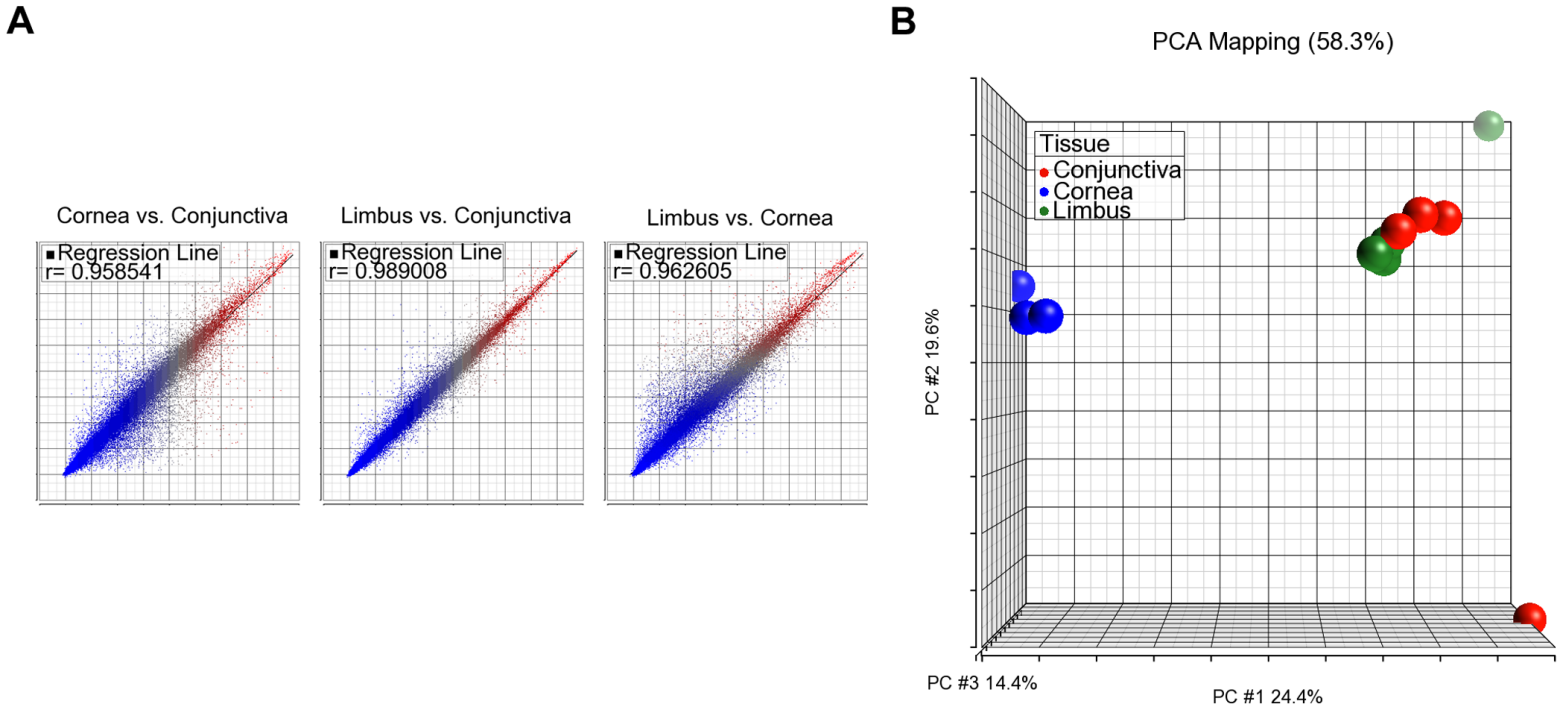
Tissue correlation of global gene expression between ocular samples. A) Pairwise scatter plots of the correlation of transcript expression between cornea, conjunctiva and limbus. High correlation was observed between the limbus and conjunctiva, r = 0.989. Lower correlation was observed between the limbus and cornea, r = 0.962, and cornea and conjunctiva, r = 0.958. B) PCA analysis between the 11 samples from the cornea, conjunctiva and limbus. Each sample was clustered into common groups based on global gene expression. High correlation was observed between the limbus (green) and conjunctiva (red) and low correlation was observed between the cornea (blue) versus the limbus and conjunctiva.

When the overall expression levels were compared among all three anatomic locations using pairwise scatter plot analysis, there was very high correlation between the limbus and conjunctiva (r = 0.989), while lower correlations between the limbus and cornea (r = 0.963) and conjunctiva and cornea (r = 0.959) were observed (Fig. 1A). Principal Component Analysis (PCA) was used to visually represent the total microarray data for each of the tissue replicates. The cornea replicates were tightly clustered together and distant from the adjacently clustered conjunctival and limbal groups (Fig. 1B). The high correlation observed between the conjunctiva and limbus in the pairwise scatter plot was consistent with the PCA results.

### Upregulated Transcripts in the Human Limbus

**Figure 2:**
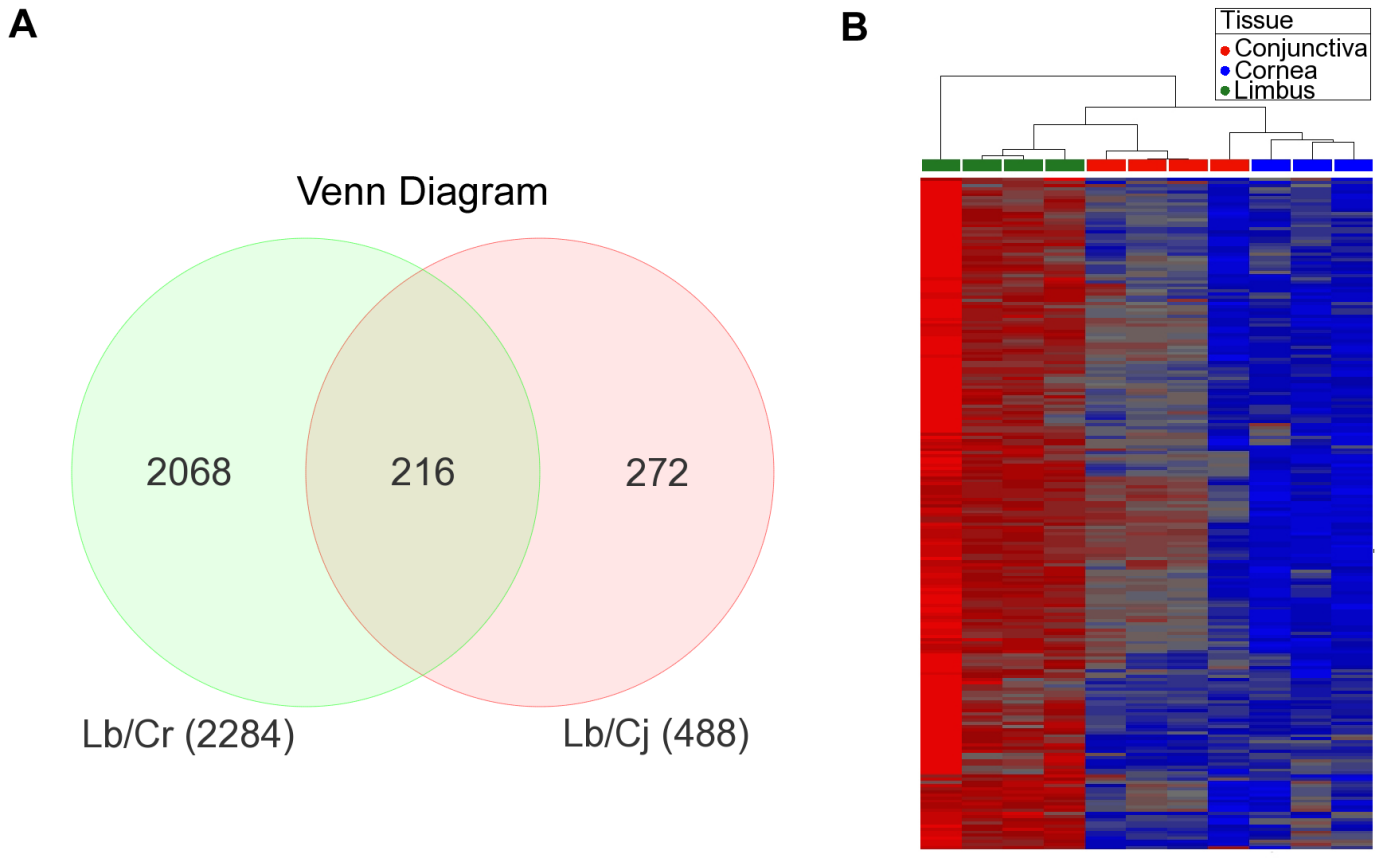
Analysis of preferential transcription in the limbus compared to the cornea and conjunctiva. A) Venn diagram of 216 preferentially expressed limbal transcripts (including unknown and overlapping probes) between the limbal over corneal and conjunctival transcripts. B) Heat map of the 216 preferentially expressed limbal transcripts (including unknown and overlapping probes) between the limbal over corneal and conjunctival transcripts categorized by anatomical tissue. Red signifies overexpression and blue signifies underexpression.

When we compared the expression levels of transcripts from the limbus to that in the cornea, 2284 transcripts were found to be upregulated in the limbus, whereas 488 transcripts were overexpressed in the comparison between the limbus and conjunctiva. Combining the data sets by utilizing a Venn diagram, we identified 216 transcripts that were differentially expressed in the limbus against those in both the cornea and conjunctiva (Fig. 2A). Of those 216 differentially expressed transcripts, 146 were known protein-encoding genes. A heatmap of the 216 transcripts from all 11 tissue samples confirms the specific expression of these transcripts in the limbus (Fig. 2B). Similar expression patterns were observed among the samples from the same anatomical location, indicative of a fairly consistent expression profile among different donors. The top expressing genes from our list of 146 upregulated limbal genes were segregated based on biological cell functions and summarized in Table 1. The biological groups were ranked according to the number of gene hits per class from highest to lowest. We identified several genes upregulated in the limbus that are involved in important biological cell processes, such as cell adhesion, proliferation, mobility, differentiation and wound healing. Furthermore, two key signaling pathways, the Wnt and TGF-β/BMP pathways, had multiple components that were present in the 146 limbal gene list.

**Table 1:** Preferentially Expressed Limbal Genes Grouped by Biological Function.

Gene Category	List Hits	Genes	P-value
cell adhesion	24	ACTN1; AEBP1; CD93; CDH11; CDH19; CNTN3; COL12A1; COL13A1; COL16A1; COL24A1; EMCN; ECM2; FERMT2; FBLN5; FN1; ITGA10; POSTN; SPP1; SRPX; SVEP1;THBS4; TNC; TNN; VCAM1	2.20E−08
wound healing	15	DARC; CFH; CDO1; EPHA3; F2R; F2RL2; FGF2; FBLN5; FN1; MAP1B; SERPINA3; SERPING1; SPP1; TIMP3; TNC	1.70E−04
cell proliferation	15	AXIN2; BMP4; CCL14/15; EDNRA; F2R; FGF18; FGF2; ID4; PRRX1; PTGER2; PTN;RBP4; TIMP2; VCAM1; ZEB1	7.40E−03
cell migration	9	DCLK1; ENPEP; FGF2; FN1; TNN; THBS4; TNN; TWIST1; VCAM1	2.60E−03
cell differentation	9	ANGPT1; BMP4; COL13A1; DCLK1; FGF2; FHL1; FRZB; NTRK3; TWIST1	1.90E−02
TGF-β signaling pathway	5	BMP4; FMOD; ID4; LTBP2; THBS4	3.00E−02
WNT signaling pathway	4	AXIN2; FRZB; FZD7; PITX2	5.90E−02

### Confirmation of Selected Upregulated Genes in the Limbus

**Figure 3:**
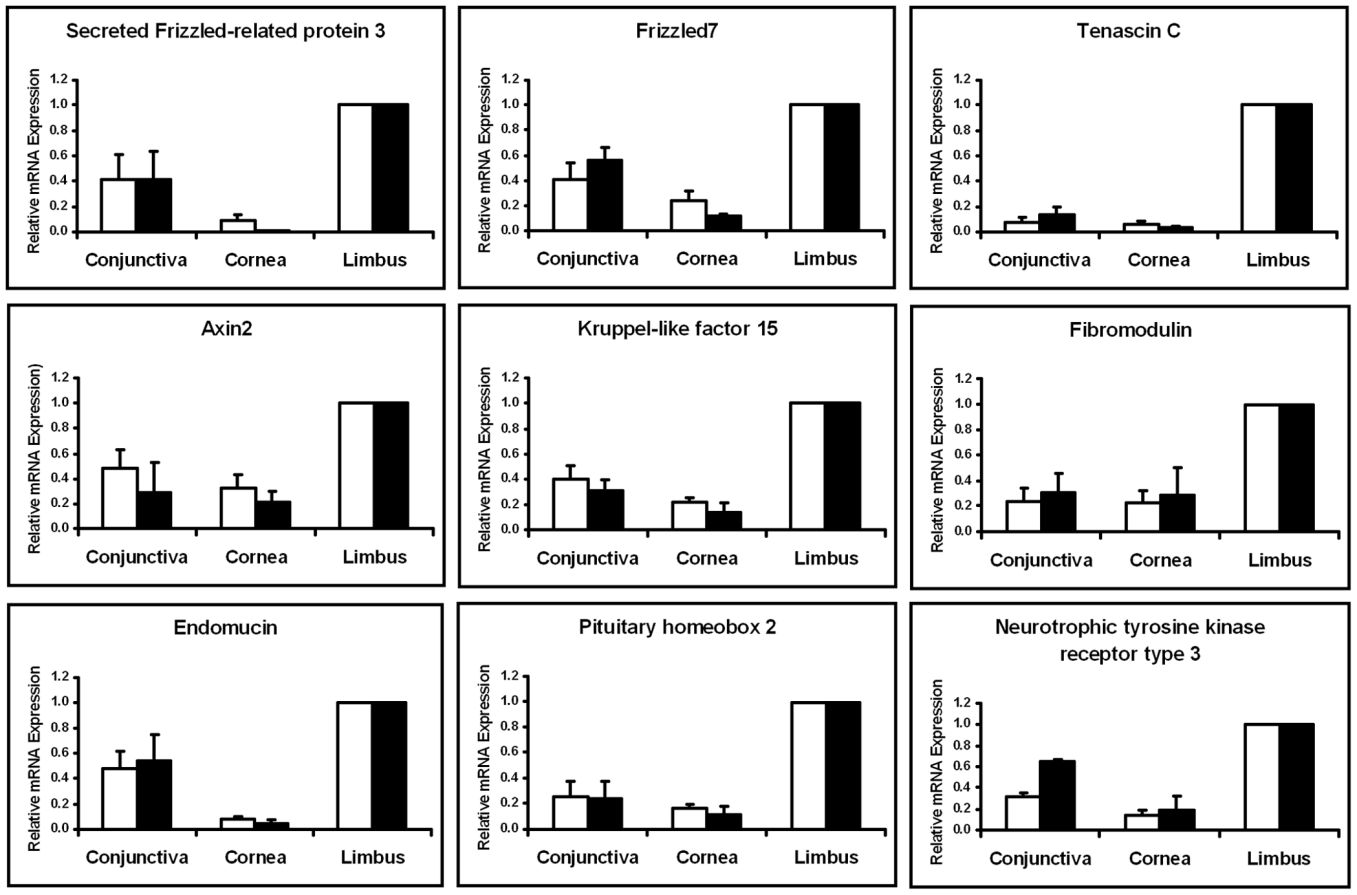
Comparison of mRNA expression levels between microarray and qRT-PCR of selected transcripts. All 9 transcripts were preferentially expressed in the limbus and minimally expressed in the cornea and conjunctiva. White bars represent the microarray results and black bars represent the qRT-PCR results. Similar expression patterns were observed between the microarray and qRT-PCR.

To further validate our microarray data, we examined nine differentially expressed limbal genes by qRT-PCR for secreted frizzled-related protein 3 (SFRP3) or frizzled-related protein (FRZB), frizzled-7 (FZD7), tenascin-C (TNC), axin-2, kruppel-like factor 15 (KLF15), fibromodulin, endomucin, pituitary homeobox 2 (PITX2) and neurotrophic tyrosine kinase receptor type 3 (TRKC). All nine genes displayed similar expression patterns in the three tissue types by microarray and qRT-PCR (Fig. 3).

In addition to analyzing the nine upregulated limbal genes from the microarray, we also compared the expression profiles of several of the known putative LSC markers such as ABCG2, K15, integrin α9, C/EBPΔ and N-cadherin. None of the markers were among the 146 limbal genes due to high marker expression in the conjunctiva (Fig. S3). However, when the relative expression was compared between the limbus and cornea only, we noted overexpression of these markers in the limbus (Fig. S3). In addition, the mature corneal epithelial marker pair, K3/12, had the highest expression in the cornea.

### Confirmation of Upregulated Limbal Transcripts at the Protein Level

**Figure 4:**
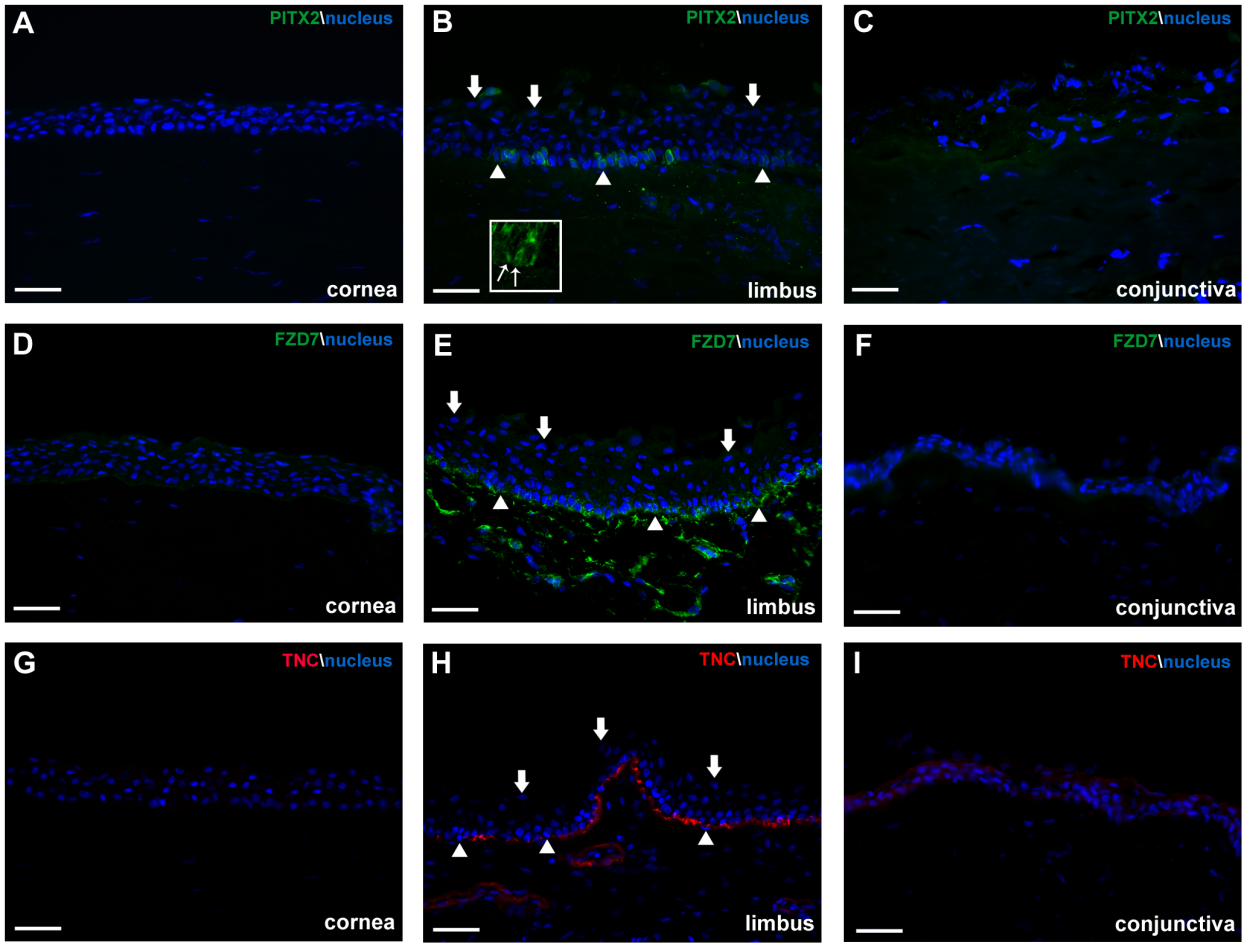
Protein expression of selected limbal transcripts in human ocular tissue. A) Minimal expression of PITX2 was observed in the cornea. B) Distinct PITX2 cytoplasmic expression was present in pockets within the basal layer and suprabasal layer of the limbal epithelium. Insert highlights both PITX2 cytoplasmic and nuclear localization in the basal epithelial cells. Thin arrows indicate cells with PITX2 nuclear localization. C) Minimal expression of PITX2 was also observed in the conjunctiva. D) Weak expression of FZD7 was detected in the basal layer of the cornea. E) Highly localized FZD7 expression was observed at the basal layer of the limbal epithelium. F) We observed low expression in the suprabasal and superficial layers of the conjunctiva for FZD7. G) Minimal expression of TNC was observed in the cornea, while distinct expression was present in the subepithelial stroma along the limbus (H). (I) We detected minor expression of TNC in the suprabasal and superficial layers of the conjunctiva. Thick arrows represent examples of superficial epithelial cells and arrowheads represent examples of basal epithelial cells in the limbus. Scale bar, 50 µm.

We next examined the spatial protein expression patterns of selected genes that were preferentially expressed in the limbus by immunohistochemistry in human ocular tissue sections. We observed specific expression of PITX2, FZD7 and TNC in the limbus. The majority of PITX2 was detected at the basal and suprabasal layer of the epithelium (Fig. 4B). Confocal microscopy confirmed that PITX2 was concentrated in the cytoplasm of basal and suprabasal epithelial cells, with a few cells containing PITX2 nuclear localization (Fig. 4B, insert). Minimal expression was observed in the cornea and the conjunctiva (Fig. 4A,C). FZD7 had distinct expression in the basal epithelial layer of the limbus (Fig. 4E) and minimal expression in the basal corneal and conjunctival epithelium (Fig. 4D,F). TNC was also specifically present in the subepithelial stroma in a linear fashion along the limbus (Fig. 4H), with very weak expression in the cornea and minor expression in the suprabasal and superficial layers of the conjunctiva (Fig. 4G, I).

### Gene Ontology and Network Pathway Analysis

**Figure 5:**
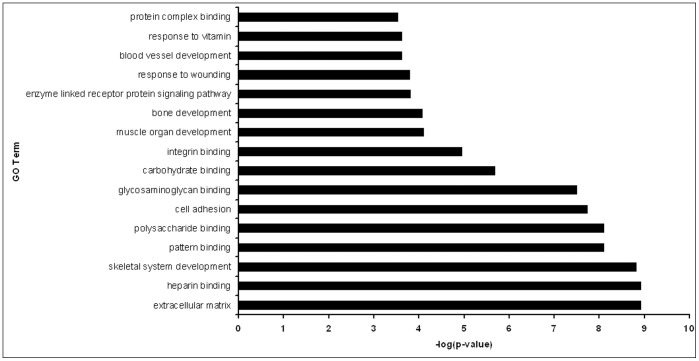
Gene ontology analysis of preferentially expressed signature limbal genes. Bar graph showing significance of enrichments terms from preferentially expressed genes in the limbal tissue.

The DAVID database was used to categorize the 146 differentially expressed limbal genes into functional groups. Figure 5 highlights the major biological functions from the list of limbal genes based on the significance (p-value) of enrichments terms. Cell adhesion and extracellular matrix associated genes have the highest significance in the GO term analysis (Fig. 5). Several collagens, cadherins and tenascins were present in the cell adhesion group, which was most likely due to the inclusion of stromal cells in the samples. Interestingly, a few developmental processes, such as bone and muscle development were observed in the GO term analysis. There were also several growth factors present, including tumor necrosis factor receptor superfamily, member 19 (TNFRS19) and chemokine (C-C motif) ligand 14, as well as fibroblast growth factor (FGF) 18 and bone morphogenetic protein (BMP) 4.

**Figure 6:**
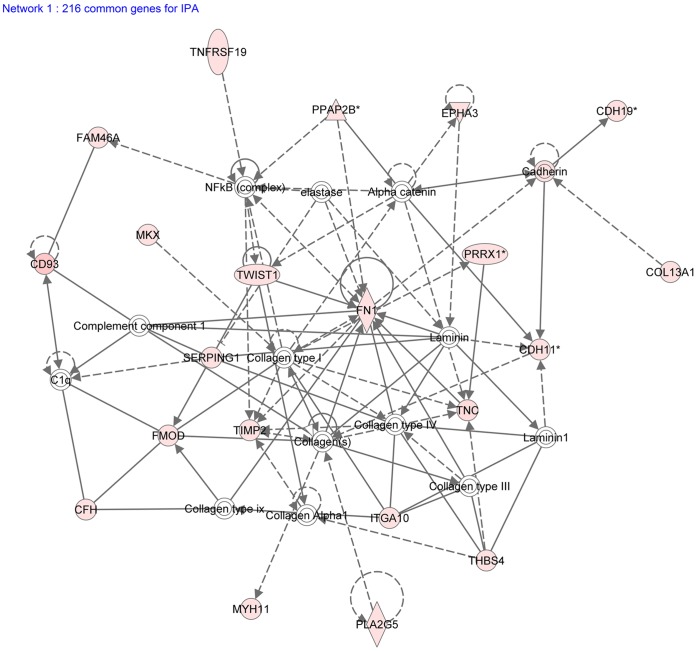
Network map of preferentially expressed signature limbal genes. The IPA network map highlights the upregulation of genes from the TGF-β pathway and extracellular matrix processes. Fibronectin-1 is centrally connected to a number of upregulated limbal genes.

We confirmed expression of several Wnt-related genes in the limbus (Table 1). These include three important Wnt signaling components, FZD7, a canonical Wnt receptor, FRZB, an inhibitor of Wnt signaling, and PITX2, a downstream target of Wnt signaling that also functions as a negative regulator in Wnt signaling [28], [29]. We identified several genes from the TGF-β/BMP signaling pathway in the human limbus. BMP4, thrombospondin 4, latent transforming growth factor beta binding protein 2, fibromodulin and intracellular inhibitor of DNA binding 4 protein were among the upregulated genes in the limbus (Table 1). Using Ingenuity Systems’ web-based software, IPA, we generated a network map from the 216 identified transcripts in the limbus (Fig. 6). We observed several upregulated genes (red) from the extracellular matrix group and TGF-β/BMP pathway. Interestingly, fibronectin-1 was connected to several other upregulated genes and centrally located in the network map. This indicates that fibronectin-1 might be a key component of the extracellular matrix in the LSC niche [30] and serves as a communication link between epithelial cells and the basement membrane.

## Discussion

A few reports have identified a number of potential limbal epithelial stem/progenitor cell markers; however neither a specific LSC marker nor specific regulatory process has yet been identified. One limitation of these previous studies is that the other neighboring tissue, the conjunctiva, was not included. As we have shown, many of the putative limbal stem cell markers were also expressed in the conjunctiva and none of the previous differential gene profiling studies has compared the limbal epithelium to both the cornea and conjunctiva. In this current study, we specifically include all three anatomical tissues, the conjunctiva, cornea and limbus to identify those transcripts that are indeed preferentially expressed in the limbal region only. We also compared our results to the three most comprehensive microarray studies to date [16], [21], [22]. Several transcripts were shared between our study and others, including BMP4, cadherin 19 (CDH19), FAM107A, FZD7, glycoprotein hormone alpha-2 (GPHA2), FRZB, serpin peptidase inhibitor, clade F (alpha-2 antiplasmin, pigment epithelium derived factor), member 1 (SERPINF1), SPARC-like protein 1 and TNFRS19. Figueira et al. detected unique gene expression of BMP4 in human fetal limbal tissue but not in the central cornea using microarrays specific to stem cell gene expression [16]. The Dua group utilized laser capture microdissection to isolate limbal and corneal epithelial regions from adult humans. They observed upregulation of both SERPINF1 and FRZB in limbal epithelial crypts compared to the corneal epithelium. In addition, they confirmed protein expression of FRZB in the basal epithelium of the limbal crypts, but not in the central cornea [21]. Finally Vereb et al. compared adult human epithelial cell sheets from the limbus and cornea and their data set had the most genes in common with our study. Using the identical U133 Plus 2.0 microarray chip, they also found CHD19, FAM107A, FZD7, GPHA2, SPARC-like protein 1 and TNFRS19 were upregulated in the limbal epithelium [22]. Detailed comparisons of our data with those published are not feasible due to the lack of complete data sets. Overall, our study identified a unique set of preferentially expressed limbal genes which highlights the significance of including both the conjunctiva and cornea.

We have previously shown that Wnt signaling is present in the limbus in vervet monkey and may play a role in human LSC processes [19], [31]. As expected, a number of Wnt signaling components were also present in the human limbal region, including FZD7, axin-2 and FRZB (Table 1). There are several FZD proteins and they act as receptors for Wnts, by transmitting both Wnt canonical and noncanonical signaling. FZD7 is expressed by other stem cells such as in embryonic stem cells (ESCs). Knockdown of FZD7 using small hairpin RNA in ESCs led to differentiation of cells at the edge of colonies compared to the control [32]. Recently, fibronectin-1 was linked to a complex composed of FZD7 and syndecan-4 in the satellite stem cells of the mouse muscle [33]. Fibronectin-1 was found to enhance the Wnt7a transduction signal and promote proliferation of satellite stem cells by coupling to the FZD7 and syndecan-4 complex. Whether FZD7 has a similar regulatory function in LSCs is unknown.

Another limbal gene and homeobox transcription factor, PITX2, has been shown to be a key regulator in ocular development. Mutations in the *PITX2* gene are associated with Axenfeld-Rieger syndrome, where malformations occur in the anterior chamber of the eye during development [34]. PITX2 is also a downstream target of Wnt signaling and can control cell proliferation [29]. Gage et al. found that there was an absence of global DKK2 expression in the eyes along with a robust increase in axin-2 expression throughout the ocular surface ectoderm in the *Pitx2* knockout mice [28]. Another study showed that the absence of DKK2 led to the transdifferentiation of corneal epithelium into a stratified keratinized epidermal epithelium, suggesting that Wnt signaling is an important regulator of corneal cell fate determination and differentiation during development in mice [35]. In *Pitx2* and *Dkk2* deficient mice, Wnt signaling is continuously activated [28], suggesting that PITX2 can negatively regulate canonical Wnt signaling through DKK2 and thus suppress local Wnt activity. In a separate study, PITX2 was found to upregulate several Wnt ligand genes and downregulate several FZD receptors in ovarian cancer cells [36]. The increase in mRNA levels of the Wnt ligands correlated with an increase in cell proliferation.

Our current study is the first to show the specific expression of PITX2 in the limbal basal epithelium in humans (Fig. 4B). The majority of PITX2 localization was cytoplasmic, but we did observe a small number of basal epithelial cells that had nuclear localization of PITX2 (Fig. 4B, insert). This might be a regulatory step of PITX2 function in limbal stem/progenitor cells. It is possible that since the endogenous Wnt activity is normally low in the limbus and PITX2 is a downstream target of Wnt signaling, this could explain why there was very little PITX2 nuclear localization. Whether PITX2 also assumes a similar function in humans as a negative regulator of Wnt signaling via DKK2 or positive regulator through Wnt ligands, is yet to be investigated.

Cell adhesion is the leading biological process in the limbus from our microarray study (Table 1). We confirmed the level of expression of three extracellular matrix proteins, TNC, endomucin and fibromodulin by qRT-PCR analysis (Fig. 3) and the protein expression of TNC specifically in the basal layer of the limbus (Fig. 4H). TNC is an extracellular matrix protein that regulates cell adhesion and migration [37]. Due to TNC’s ability to bind to both extracellular matrix proteins and cell surface receptors and its presence in other stem cell niches [38], it is possible that this protein is a LSC niche factor as well. Endomucin is a cell membrane bound extracellular matrix-associated protein, where it can block cell and extracellular matrix interactions [39]. Its presence in the limbus might modulate regulatory signals from the extracellular matrix, by inhibiting the interactions between extracellular matrix proteins and LSCs. Fibromodulin belongs to a family of small leucine-rich proteoglycans/glycoproteins. In mice, it has been shown to be temporally expressed in the limbus during the postnatal development of the anterior eye [40]. Fibromodulin is also expressed in epidermal keratinocytes and functionally it can sequester TGF-β growth factors in the extracellular matrix and hence can act as a regulatory step [41], [42]. It is possible that these membrane proteins control the presentation of sequestered growth factors in the limbus. Only upon changes in the microenvironment, these adhesion molecules might influence the interactions between LCSs and the extracellular matrix and allow for contact between the growth factors and LSCs to regulate cell proliferation and differentiation.

In addition to Wnt signaling, several components of the TGF-β/BMP pathway were also present in the limbus. This signaling pathway has been implicated as a growth inhibitor of stem cells [43]. Several TGF-β/BMP ligands and receptors have been previously observed in the limbus, including TGF-β1, TGF-β2, TGF-β receptor types I (RI) and II (RII) [44]–[46]. Other BMP ligands and receptors have been shown to be expressed in ex vivo and cultured epithelium [47].

BMP4 is an important differentiation factor in the TGF-β/BMP pathway and was shown to inhibit intestinal stem cell self-renewal through cross talk with canonical Wnt signaling, while others have shown that BMP signaling can restrict hair follicle stem cell proliferation and maintain stem cells in a quiescent state [48], [49]. Studies in mouse bone marrow mesenchymal stem cells (MSCs) have shown that BMP2 represses canonical Wnt signaling and decreases MSC proliferation through interactions between Dishevelled1 and Smad1, thereby inhibiting β-catenin nuclear localization [50]. The function of BMP4 in LSC maintenance is yet to be determined.

In summary, we have identified a new group of genes that are preferentially expressed in the human limbus compared to both the cornea and conjunctiva. Our findings could aid in the identification of LSC niche factors that may regulate LSCs. Additionally, the identification of specific components from the Wnt and TGF-β/BMP pathways warrants new investigations into their potential role in the regulation of LSC self-renewal and differentiation.

## Supporting Information

Figure S1 **Total RNA analysis on ocular tissue samples.** 11 Total RNA samples were analyzed using a Agilent 2100 Bioanalyzer. Graphs for each sample had two distinct peaks at 18 S and 28 s. Nanogels for each sample also had two distinct bands at 18 S and 28 s. Missing cornea sample D3R was due to lack of RNA during the isolation. Abbreviations: D (Donor), R (Right), L (Left).
(TIF)

Figure S2 **Expression levels of selected cornea, conjunctiva and limbal markers.** mRNA expression levels were consistent with expected expression patterns by qRT-PCR. K12 expression was highest in the cornea. K15 was expressed in both the limbus and conjunctiva. Mucin-5AC expression was highest in the conjunctiva. Abbreviations: K (cytokeratin).
(TIF)

Figure S3 **Expression levels of selected putative limbal stem cell markers and mature corneal epithelial markers.** mRNA expression levels of several putative limbal stem markers were expressed in both limbal and conjunctival tissue through microarray analysis. K12 was expressed in the cornea and limbus. Abbreviations: (ATP-binding cassette sub-family G member 2), ITGA9 (Integrin alpha-9), CDH2 (Cadherin-2 or neural cadherin), (C/EBPΔ) CCAAT/enhancer-binding protein delta, K (cytokeratin).
(TIF)

Table S1 List of qRT-PCR Primers.
(DOC)

Table S2 List of Primary and Secondary Antibodies.
(DOC)
