# Supplementary figures and images for: Preferential Biological Processes in the Human Limbus by Differential Gene Profiling
Source: PLoS One. 2013 Apr 22;8(4):e61833. doi: 10.1371/journal.pone.0061833 (PMC3632514; doi:10.1371/journal.pone.0061833)

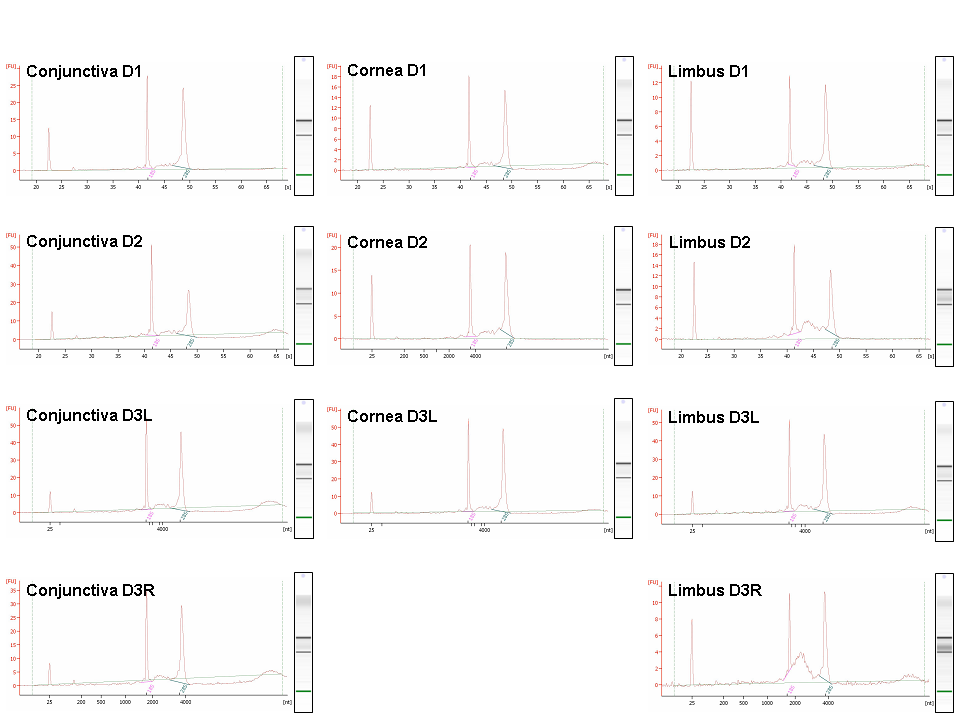

Supplement: Figure S1 — Total RNA analysis on ocular tissue samples. 11 Total RNA samples were analyzed using a Agilent 2100 Bioanalyzer. Graphs for each sample had two distinct peaks at 18 S and 28 s. Nanogels for each sample also had two distinct bands at 18 S and 28 s. Missing cornea sample D3R was due to lack of RNA during the isolation. Abbreviations: D (Donor), R (Right), L (Left). (TIF) [file pone.0061833.s001.tif]

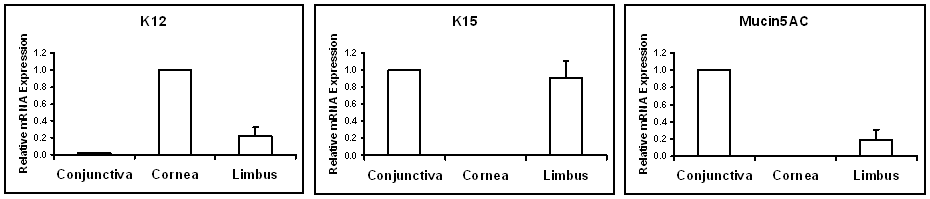

Supplement: Figure S2 — Expression levels of selected cornea, conjunctiva and limbal markers. mRNA expression levels were consistent with expected expression patterns by qRT-PCR. K12 expression was highest in the cornea. K15 was expressed in both the limbus and conjunctiva. Mucin-5AC expression was highest in the conjunctiva. Abbreviations: K (cytokeratin). (TIF) [file pone.0061833.s002.tif]

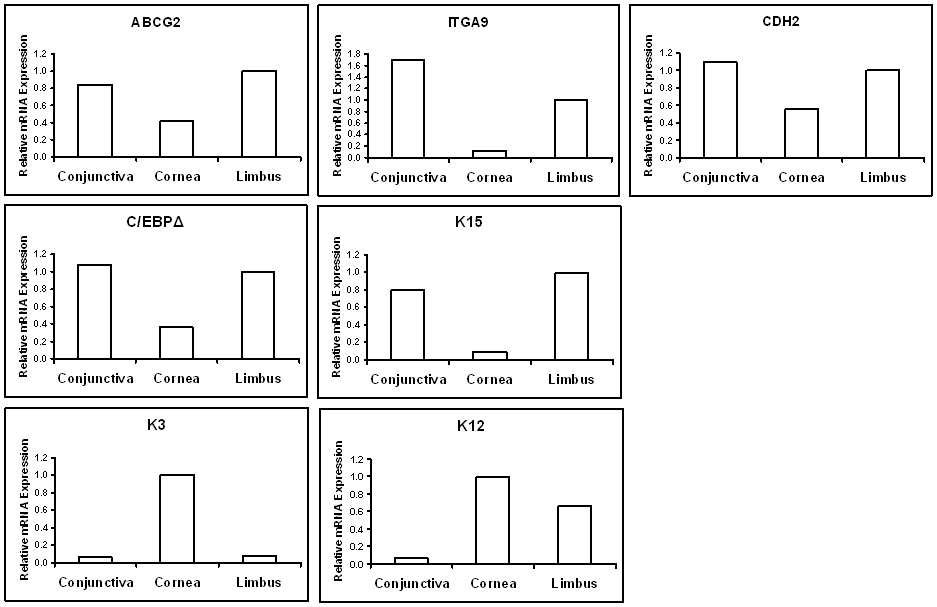

Supplement: Figure S3 — Expression levels of selected putative limbal stem cell markers and mature corneal epithelial markers. mRNA expression levels of several putative limbal stem markers were expressed in both limbal and conjunctival tissue through microarray analysis. K12 was expressed in the cornea and limbus. Abbreviations: (ATP-binding cassette sub-family G member 2), ITGA9 (Integrin alpha-9), CDH2 (Cadherin-2 or neural cadherin), (C/EBPΔ) CCAAT/enhancer-binding protein delta, K (cytokeratin). (TIF) [file pone.0061833.s003.tif]
