# Supplementary material for: Preferential Biological Processes in the Human Limbus by Differential Gene Profiling
Source: PLoS One. 2013 Apr 22;8(4):e61833. doi: 10.1371/journal.pone.0061833 (PMC3632514; doi:10.1371/journal.pone.0061833)
Supplement: Table S1 — List of qRT-PCR Primers. (DOC) [file pone.0061833.s004.doc]

| **Table S1. qRT-PCR Primers** | | |
| --- | --- | --- |
| **Gene** | **Direction** | **Primer Sequence** |
| K12 | Forward | CCAGGTGAGGTCAGCGTAGAA |
|  | Reverse | CCTCCAGGTTGCTGATGAGC |
| K15 | Forward | ACCACCACATTTCTGCAAACT |
|  | Reverse | AGCTGAGATACTTCGGCTTCC |
| MUC5AC | Forward | CAGCCACGTCCCCTTCAATA |
|  | Reverse | ACCGCATTTGGGCATCC |
| AXIN2 | Forward | AGCCAAAGCGATCTACAAAAGG |
|  | Reverse | GGTAGGCATTTTCCTCCATCAC |
| FRZB | Forward | TCCCGAGGCCATCGTTACT |
|  | Reverse | AGGCTTACATTTACAGCGTTCAC |
| FZD7 | Forward | CCGTACCACGGAGAGAAGG |
|  | Reverse | GCGGAGTTCGGGAGAACAC |
| EMCN | Forward | CAGCAACCAGCCGGTCTTATT |
|  | Reverse | GCACATTCGGTACAAACCCAC |
| FMOD | Forward | GAGACCTACGAGCCTTACCC |
|  | Reverse | ATGGCCGTGAGGAAGTTGG |
| KLF15 | Forward | GTTGGGTATCTGGGTGATAGGC |
|  | Reverse | TGAGAGTCGGGACTGGAACAG |
| NTRK3 | Forward | TGGCTGGACTATGTGGGCT |
|  | Reverse | CCCATTGCTGTTCCCTGAATC |
| PITX2 | Forward | CACCATCCCCAGCCGTTAG |
|  | Reverse | GCTTCTTCTTAGACGGGTCCTC |
| TNC | Forward | TGCTCTGAGCCCGAATGTC |
|  | Reverse | TGGGCAGATTTCACGGCTG |
| GAPDH | Forward | GGCTGAGAACGGGAAGCTTGTCAT |
|  | Reverse | CAGCCTTCTCCATGGTGGTGAAGA |
