# Supplementary material for: Preferential Biological Processes in the Human Limbus by Differential Gene Profiling
Source: PLoS One. 2013 Apr 22;8(4):e61833. doi: 10.1371/journal.pone.0061833 (PMC3632514; doi:10.1371/journal.pone.0061833)
Supplement: Table S2 — List of Primary and Secondary Antibodies. (DOC) [file pone.0061833.s005.doc]

| **Table S2. Primary and Secondary Antibodies** | | | |
| --- | --- | --- | --- |
| **Protein** | **Company** | **Host** | **Species Reactivity** |
| Frizzled-7 (FZD7) | R&D Systems (Minneapolis, MN) | rat | human/mouse |
| Paired-like homeodomain 2 (PITX2) | Santa Cruz Biotechnology (Santa Cruz, CA) | goat | human |
| Tenascin-C (TNC) | Santa Cruz Biotechnology (Santa Cruz, CA) | rabbit | human |
| Alexa Fluor 488 IgG | Invitrogen, Carlsbad, CA | donkey | goat |
| Alexa Fluor 488 IgG | Invitrogen, Carlsbad, CA | donkey | rat |
| Alexa Fluor 488 IgG | Invitrogen, Carlsbad, CA | donkey | rabbit |
